# Three synchronous primary carcinomas in a patient with HNPCC associated with a novel germline mutation in *MLH1*: Case report

**DOI:** 10.1186/1477-7819-7-94

**Published:** 2009-12-08

**Authors:** Cristian D Valenzuela, Harvey G Moore, William C Huang, Elsa W Reich, Herman Yee, Harry Ostrer, H Leon Pachter

**Affiliations:** 1 Department of Surgery, NYU Langone Medical Center, New York, USA; 2 Department of Urology, NYU Langone Medical Center, New York, USA; 3 Human Genetics Program, NYU Langone Medical Center, New York, USA; 4 Department of Pathology, NYU Langone Medical Center, New York, USA

## Abstract

**Background:**

*MLH1 *is one of six known genes responsible for DNA mismatch repair (MMR), whose inactivation leads to HNPCC. It is important to develop genotype-phenotype correlations for HNPCC, as is being done for other hereditary cancer syndromes, in order to guide surveillance and treatment strategies in the future.

**Case presentation:**

We report a 47 year-old male with hereditary nonpolyposis colorectal cancer (HNPCC) associated with a novel germline mutation in *MLH1*. This patient expressed a rare and severe phenotype characterized by three synchronous primary carcinomas: ascending and splenic flexure colon adenocarcinomas, and ureteral carcinoma. Ureteral neoplasms in HNPCC are most often associated with mutations in *MSH2 *and rarely with mutations in *MLH1*. The reported mutation is a two base pair insertion into exon 10 (c.866_867insCA), which results in a premature stop codon.

**Conclusion:**

Our case demonstrates that HNPCC patients with *MLH1 *mutations are also at risk for ureteral neoplasms, and therefore urological surveillance is essential. This case adds to the growing list of disease-causing MMR mutations, and contributes to the development of genotype-phenotype correlations essential for assessing individual cancer risk and tailoring of optimal surveillance strategies. Additionally, our case draws attention to limitations of the Amsterdam Criteria and the need to maintain a high index of suspicion when newly diagnosed colorectal cancer meets the Bethesda Criteria. Establishment of the diagnosis is the crucial first step in initiating appropriate surveillance for colorectal cancer and other HNPCC-associated tumors in at-risk individuals.

## Background

Colorectal cancer (CRC) is currently the third leading cause of cancer and cancer-related deaths in the United States, estimated to be responsible for 10% of new cancer cases and 9% of cancer deaths in 2008 [1]. Hereditary nonpolyposis colorectal cancer (HNPCC), or Lynch syndrome, is the most common heritable CRC syndrome [2], accounting for approximately 5% of all CRC cases. Transmitted in an autosomal dominant fashion, HNPCC is associated with tumorigenesis caused by mutations in one of several genes involved in DNA mismatch repair (MMR) [3]. About 90% of HNPCC cases are associated with mutations in *MLH1 *(OMIM #120436) or *MSH2 *(OMIM #609309), and others are associated with mutations in *MSH6*, *PMS1*, *PMS2*, and *MLH3 *[4]. Reported MMR mutations have been catalogued in three online databases that document the full spectrum of mutations and phenotypes associated with HNPCC [5-7].

In HNPCC, as in various other cancer syndromes, a somatic mutation or epigenetic event is needed as the "second hit" to disable the functional, wild-type allele [8]. Abolition of MMR raises the basal rate of mutation and leads to microsatellite instability (MSI), a tissue marker of defective mismatch repair [9]. This increased level of mutagenesis is most likely to affect tissues that undergo a high rate of cell division, such as the colonic epithelium [10]. Carriers of MMR mutations have up to an 82% lifetime risk of developing CRC [11,12]. Aside from demonstrating an MMR mutation, patients may be clinically diagnosed with HNPCC if their family cancer history meets the Amsterdam Criteria II, which encompasses CRC and associated extracolonic sites [13].

HNPCC also carries the risk of developing cancer at various other sites. The most clinically significant extracolonic cancer associated with HNPCC is endometrial cancer, for which women have a 60% lifetime risk [11]. Compared to the general population, patients with HNPCC are 14-fold more likely to develop urothelial cell carcinoma of the upper urinary tract, corresponding to a cumulative lifetime risk of 2.6%-4.0% [11,14]. We report the case of a 47-year old man with a rare presentation of two distinct colorectal adenocarcinomas and a synchronous ureteral neoplasm. A definitive diagnosis of HNPCC was only established post-operatively following identification of a not previously reported germline mutation in *MLH1*.

## Case presentation

A 47-year old man of Puerto Rican descent presented with recent gross hematuria and a history of lower abdominal pain of 1-2 years duration. Other comorbidities included hypertension and Type II diabetes mellitus. He had a 20-year history of heavy smoking. Colonoscopy three years prior to presentation revealed no evidence of adenomatous polyps or colorectal cancer.

**Figure 1:**
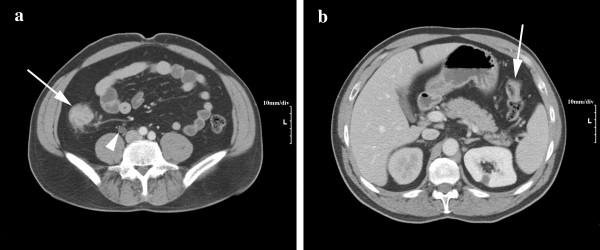
**(a) CT scan with PO/IV contrast suggested a large tumor of the ascending colon (arrow)**. Enlarged pericolic lymph nodes are suggestive of metastases. Also shown is right-sided hydroureteronephrosis caused by the mid-ureteral tumor (arrowhead). (b) The colon tumor at the splenic flexure tumor is visible on a different cut (arrow).

Urine cytology revealed the presence of numerous red blood cells, as well as atypical urothelial cells. A subsequent CT scan revealed a mass in the mid-right ureter suggestive of a ureteral neoplasm (Fig. 1a). A ureteroscopic biopsy revealed urothelial mucosa with papillary architecture, suspicious for invasive urothelial cell carcinoma. Subsequent colonoscopy revealed synchronous tumors in the ascending colon and splenic flexure (Fig. 1a, b), and biopsies confirmed both lesions were adenocarcinomas. A two-centimeter flat lesion was also identified in the transverse colon, and biopsy was consistent with a flat adenoma with focal high-grade dysplasia.

The three tumors were resected in a combined operation, and the patient recovered from surgery without complication. The urothelial cell carcinoma was high-grade, pT3 N2 Mx, Stage IV, and the more advanced colon adenocarcinoma was moderately differentiated, pT3 N1 Mx, Stage IIIB. The patient received adjuvant chemotherapy consisting of cisplatin and taxol combined with radiotherapy, and is disease-free at the time of publication.

**Figure 2:**
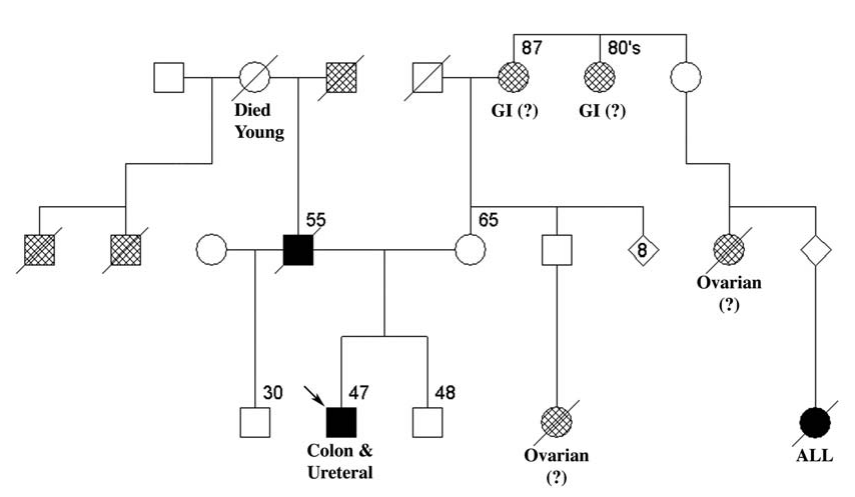
**The patient (proband) is indicated by an arrow**. Age of diagnosis or death is indicated if known. Diamonds indicate a group of siblings of unknown sex. Solid boxes indicate a diagnosis of colon cancer, and crosshatched boxes indicate cancer of non-colorectal origin. A diagonal strikethrough indicates a deceased individual. Unconfirmed diagnoses of individuals with extracolonic cancer are noted. Unaffected members of the extended family have been omitted for conciseness. One family member died in childhood from acute lymphoblastic leukemia (ALL).

A comprehensive family history was taken and a formal pedigree was assembled (Fig. 2). The patient's father died of colon cancer shortly after diagnosis at age 55, and three other paternal family members had cancer in an unknown site. The patient's paternal grandmother died at an early age of unknown causes. Four maternal family members had cancer in unconfirmed sites, and one had acute lymphoblastic leukemia.

**Figure 3:**
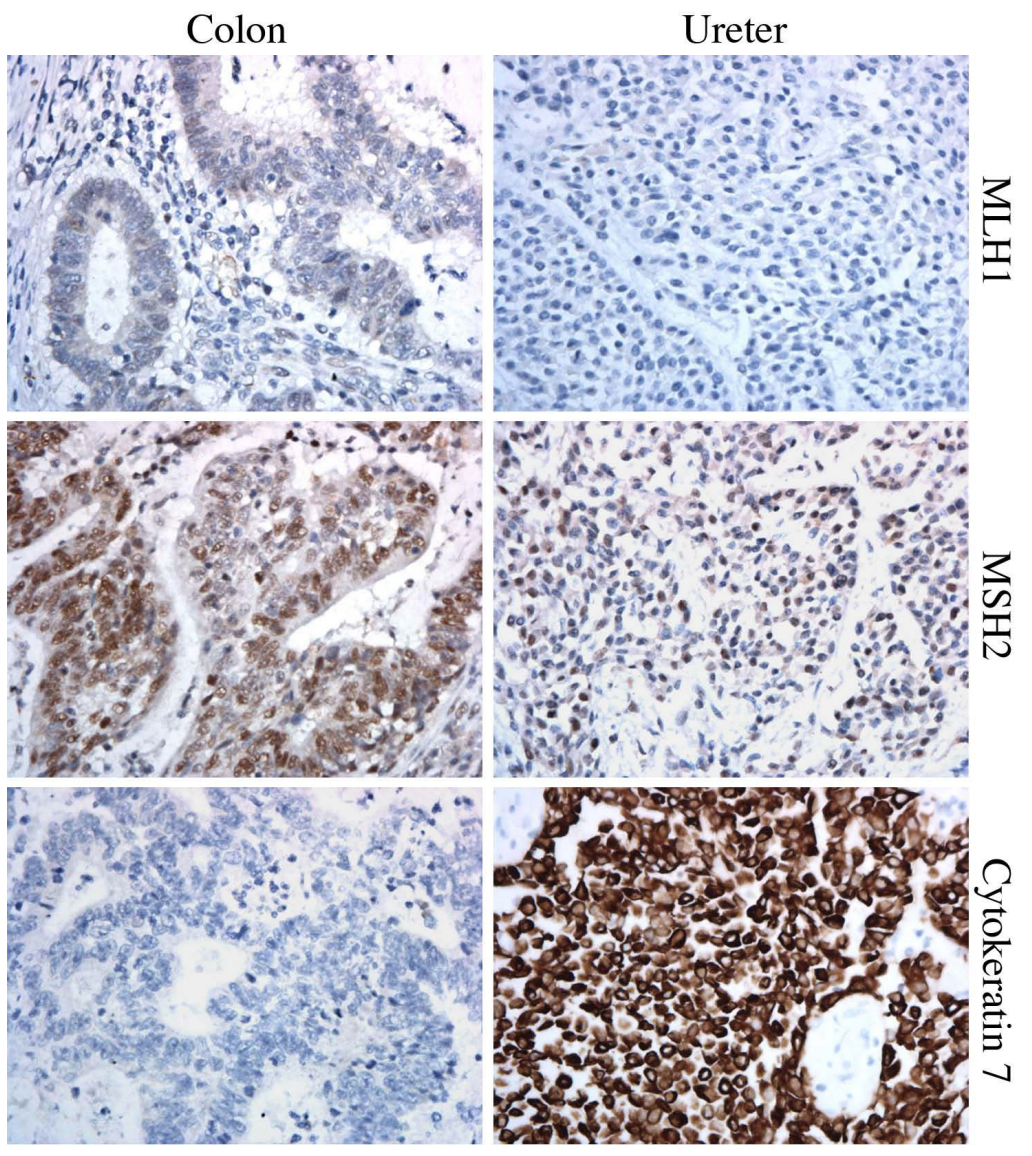
**Three immunohistochemical stains (40×) from the patient's ureteral tumor and a selected colon tumor**. The colon and ureteral cancers stained negative for MLH1 and positive for MSH2. Both colon tumors stained negative for cytokeratin 7 and positive for cytokeratin 20, whereas the ureteral tumor stained positive for both cytokeratins, demonstrating a distinct organ of origin for each tumor type and thus discounting the possibility that either tumor type is a metastasis of the other. Data not shown for positive controls.

Although the patient's family history did not satisfy Amsterdam Criteria II, the early age-of-onset and presence of synchronous tumors fulfilled the Revised Bethesda Criteria [15]. Immunohistochemistry (IHC) on surgical specimens was as follows: MLH1 stained negative and MSH2 stained positive in all tumors (Fig. 3), strongly suggesting a mutation in *MLH1*. Differential staining for cytokeratins 7 and 20 confirmed distinct histological origin of the ureteral and colon tumors (Fig. 3). Accordingly, the patient was referred for genetic counseling and germline mutation testing.

**Figure 4:**
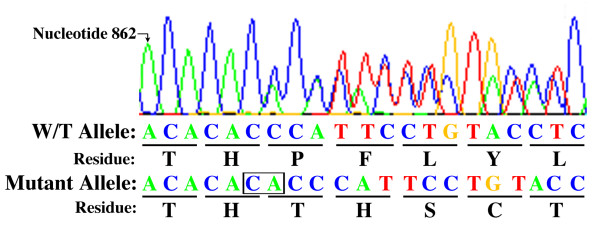
**Sequence data pertaining to the novel *MLH1 *mutation found in the proband**. A two-nucleotide 5'-CA-3' insertion (box) between base pair positions 866 and 867 leads to nonsense coding of amino acid residues and predicted truncation of the protein product (c.866_867insCA). Note: due to the palindromic nature of the insertion site, the insertion may also be classified as a 5'-AC-3' insertion between base pairs 867 and 868.

PCR amplification and sequencing on a peripheral blood sample identified the novel c.866_867insCA *MLH1 *insertion into exon 10 (Fig. 4). This frameshift mutation is predicted to produce a truncated, nonfunctional protein product due to a premature stop codon within exon 10 at amino acid residue 297. Review of the available databases revealed this specific mutation had not been previously reported.

## Discussion

There are at least 2200 total variants in the six known MMR genes, with at least 323 of these reported to be disease-causing [6]. However, unlike familial adenomatous polyposis, very little is known about phenotypic variation as a function of the MMR gene involved or the location and nature of the specific MMR gene mutation. Hence, it is important to report novel MMR mutations in order to develop genotype-phenotype correlations and add to the mutation databases that serve as a reference for genetic diagnosis.

The history of CRC in the patient's father suggests paternal origin of the *MLH1 *mutation. However, the origin of this mutation cannot be definitively determined, given that affected paternal family members are deceased and that the patient's mother declined genetic testing. Furthermore, it remains possible that a *de novo *mutation may have occurred in the proband. Nevertheless, the patient's at-risk siblings have been advised to undergo colonoscopy, evaluation of the upper urinary tracts and to seek genetic counseling.

The American Cancer Society (ACS) recommends annual or biennial colonoscopy for patients diagnosed with HNPCC by clinical criteria or genetic testing [16]. This patient's family history did not fulfill Amsterdam Criteria, and he was therefore not categorized as having HNPCC, but rather as having increased risk for CRC given a first-degree relative affected by CRC at age < 60. Thus, the patient followed the appropriate ACS screening guidelines: colonoscopy every 5 years starting at age 40. Although the revised criteria (Amsterdam Criteria II) are very specific and more sensitive than the original Amsterdam Criteria, there are still a substantial number of mutation-positive cases in which the family cancer history does not satisfy either set of Amsterdam Criteria. Limitations to obtaining an accurate family history include unavailability of original patient records, unrecognized infidelity within the family, and inaccurate patient recall. Indeed, a recent prospective population-based study found that CRC patients being evaluated for HNPCC using the Amsterdam Criteria were considerably inaccurate when recalling malignancies of family members: false-negative and false-positive rates were 39% and 21%, respectively [17]. This case illustrates the consequences of failing to recognize a hereditary CRC syndrome, despite following appropriate surveillance guidelines. Thus, recognition of newly diagnosed CRC patients that meet Bethesda Criteria should lead to evaluation with IHC and MSI testing of the tumor, as well as possible germline testing, despite a family history that does not satisfy Amsterdam Criteria. If a diagnosis of HNPCC is established, at-risk family members can be offered genetic testing and begin appropriate surveillance for HNPCC-associated tumors.

HNPCC patients have an elevated lifetime risk (approximately 3-4%) of upper urinary tract carcinoma [11,14,18]. A recent Dutch study found that patients with *MSH2 *mutations have a significantly higher risk of upper urinary tract cancer (12% by age 70, P = < 0.05) compared to patients with *MLH1 *mutations, who did not have an increased risk compared to the general population [19]. Similarly, a British study reported 13 ureteral cancers in 130 HNPCC patients; all were associated with *MSH2 *mutations [20]. In a Danish study, 11 of 12 ureteral cancers were attributable to *MSH2 *mutations (one was associated with a *MLH1 *mutation) [21]. Taken together, these studies suggest that the risk of ureteral cancer is elevated in patients with *MSH2 *mutations. Our case highlights the fact that ureteral cancer is also possible in individuals harboring *MLH1 *mutations. Others have reported similar findings [18]. Further study is necessary to determine whether surveillance recommendations for urinary tract cancer in HNPCC should be stratified by the MMR gene involved.

Specific histological characteristics have been found for urothelial cell carcinomas associated with HNPCC. Papillary architecture in urothelial cell carcinoma has a sensitivity and specificity of 70% and 78%, respectively, for predicting MSI [22]. Furthermore, IHC staining detects the loss of an MMR protein in 78-87% of upper urothelial cancers with demonstrated MSI [23,24], most commonly MSH2 and MSH6 [25]. Consistent with our patient, in HNPCC patients with upper tract urothelial cancers staining negative for at least one MMR protein, extracolonic tumors nearly always demonstrated the same MMR staining profile [25].

Unfortunately, a satisfactory screening test for ureteral cancer does not currently exist. Although annual urine cytology is generally recommended for patients with HNPCC, this test was recently found to have indeterminate sensitivity (29%) for detecting urinary tract cancer in the setting of HNPCC [21]. In HNPCC kindreds with a history of ureteral cancer or high-risk mutations, more aggressive screening may be indicated. In these patients, routine urinalysis and cytology may be combined with more contemporary methods of evaluating the upper urinary tract including cystoscopy-ureteroscopy, intravenous pyelography, ultrasonography, and CT or MR urography [14]. If suspicion of malignancy remains after cytology and imaging, FISH (fluorescence in situ-hybridization) assays may be useful: they can detect upper urothelial cell carcinomas with a sensitivity of 33% for Grade 1 and 100% for Grade 2 [26]. The two newly-approved commercial FISH assays promise to offer vast improvements over traditional cytology for detection of upper and lower urinary tract carcinomas of all grades [26].

## Conclusion

In patients with a family history characterized by multiple cases of CRC or extracolonic HNPCC-associated tumors, the possibility of HNPCC must be considered so that appropriate surveillance of at-risk individuals can be instituted. Patients presenting with ureteral cancer, particularly when MSI or suggestive histology is present, should be referred for colonoscopy as well as genetic risk assessment and possible germline testing. Conversely, in patients with known HNPCC, ureteral cancer surveillance is essential. Urine cytology alone appears to be ineffective; accuracy may be improved with the addition of cross-sectional imaging, ureteroscopic evaluation and FISH analysis. Reporting of novel disease-causing mutations, including information about ethnicity, is important for establishing genotype-phenotype correlations. It is anticipated that well-defined genotype-phenotype correlations will facilitate tailoring of surveillance strategies in the future.

## Consent

Written informed consent was obtained from the patient for publication of this case report and accompanying images. A copy of the written consent is available for review by the Editor-in-Chief of this journal.

## Competing interests

The authors have no commercial or financial associations that might create a conflict of interest with the information presented in this manuscript.

## Authors' contributions

CDV analyzed the data and wrote the first draft of the manuscript. HGM and HLP are attending surgeons who conceived the case report. WCH is the attending urologist who researched urologic tumors in HNPCC. EWR is the genetic counselor and HO is the medical geneticist who analyzed the patient's pedigree along with genetic sequencing data. HY is the attending pathologist who collected and prepared the histological figures. CDV, HGM, WCH, EWR, HY, HO, and HLP have been involved in drafting the manuscript and revising it critically for important intellectual content. All authors have read and approved the final manuscript.
